# Therapeutic efficacy of combined blockade of CTLA-4 +/- PD-L1 +/- IDO is associated with re-activation of T cells directly within the tumor microenvironment

**DOI:** 10.1186/2051-1426-1-S1-O8

**Published:** 2013-11-07

**Authors:** Stefani Spranger, Thomas Gajewski

**Affiliations:** 1 Pathology, University of Chicago, Chicago, IL, USA

## 

A major subset of human cancers is associated with CD8+ T cell infiltration yet tumor rejection fails due to the dominant effects of multiple immune inhibitory mechanisms. These include expression of the inhibitory receptors CTLA-4 and PD-1 by T cells and expression of the tryptophan-catabolizing enzyme indoleamine-2,3-dioxygenase (IDO) within the tumor microenvironment. It has been anticipated, therefore, that combined blockade of two or more inhibitory pathways may be necessary for maximal therapeutic benefit. Using the B16.SIY transplantable tumor model, we found that administration of doublets of αCTLA-4, αPD-L1 or an IDOi led to strikingly effective immune-mediated tumor control in vivo. We examined whether the potent therapeutic effect of these combinations might be attributed to increased priming versus restored function of T cells within the tumor microenvironment. Analysis of SIY-specific T cell responses in the tumor-draining lymph node or spleen early following therapy revealed only a minimal elevation with the doublets. However, within the tumor-microenvironment, each of the effective combinations was associated with markedly augmented proliferation and IL-2 production by tumor-infiltrating CD8+ T cells. No major depletion of Tregs was detected in the tumor site. In order to assess whether this increased function of tumor-infiltrating lymphocytes (TILs) was intrinsic to those cells or required new T cell entry, the S1P inhibitor FTY720 was utilized. Administration of FTY720 resulted in near total loss of circulating T cells. However, the restoration of IL-2 production and proliferation of CD8+ TILs was still observed. At later times points, after tumor rejection was proceeding, increased frequencies of SIY-specific T cells were observed in the circulation. αCTLA-4 plus αPD-L1 treatment also resulted in significant tumor control in the BrafV600E/PTEN-/- genetic tumor model. Our results suggest that combinatorial immunotherapies with potent synergy share the property of augmenting IL-2 production and proliferation by TILs directly within the tumor. These are attractive combination therapies for clinical translation, and examination of TIL proliferation should be pursued as a pharmacodynamic biomarker in the clinic.

